# Astrocytes in the nucleus of the solitary tract are activated by low glucose or glucoprivation: evidence for glial involvement in glucose homeostasis

**DOI:** 10.3389/fnins.2013.00249

**Published:** 2013-12-20

**Authors:** David H. McDougal, Gerlinda E. Hermann, Richard C. Rogers

**Affiliations:** Laboratory for Autonomic Neuroscience, Pennington Biomedical Research CenterBaton Rouge, LA, USA

**Keywords:** astrocytes, calcium imaging, 2-deoxy-D-glucose, solitary nucleus, vago-vagal reflex, glucose homeostasis, hypoglycemia

## Abstract

Glucose homeostasis is maintained through interplay between central and peripheral control mechanisms which are aimed at storing excess glucose following meals and mobilizing these same stores during periods of fasting. The nucleus of the solitary tract (NST) in the dorsal medulla has long been associated with the central detection of glucose availability and the control of glucose homeostasis. Recent evidence has emerged which supports the involvement of astrocytes in glucose homeostasis. The aim of the present study was to investigate whether NST-astrocytes respond to physiologically relevant decreases in glucose availability, *in vitro*, as well as to the presence of the glucoprivic compound 2-deoxy-D-Glucose. This report demonstrates that some NST-astrocytes are capable of responding to low glucose or glucoprivation by increasing cytoplasmic calcium; a change that reverses with restoration of normal glucose availability. While some NST-neurons also demonstrate an increase in calcium signaling during low glucose availability, this effect is smaller and somewhat delayed compared to those observed in adjacent astrocytes. TTX did not abolish these hypoglycemia mediated responses of astrocytes, suggesting that NST-astrocytes may be directly sensing low glucose levels as opposed to responding to neuronal detection of hypoglycemia. Thus, chemodetection of low glucose by NST-astrocytes may play an important role in the autonomic regulation of glucose homeostasis.

## Introduction

Glucose is the primary energy source for cellular metabolism. Therefore, the maintenance of appropriate serum glucose levels is critical for organismal survival. Glucose homeostasis is maintained through interplay between central and peripheral control mechanisms which are aimed at storing excess glucose following meals and mobilizing these same stores during periods of fasting. Central control of glucose homeostasis is accomplished by a distributed system of glucosensing elements, integrative neural networks which process signals from these elements, and pre-autonomic nuclei which control motor outflow and are modulated through inputs from these neural integrators (Watts and Donovan, 2010).

A principal site for neural integration of glucose availability is the nucleus of the solitary tract (NST), located in the dorsal medulla. Cells within NST have unique access to serum glucose levels due to the large numbers of fenestrated capillaries present in the nucleus (Gross et al., 1990) as well as its close apposition to the area postrema, a circumventricular organ (Cottrell and Ferguson, 2004). A subset of NST neurons have been shown to respond to physiologically relevant changes in extracellular glucose availability both *in vivo* (Yettefti et al., 1995, 1997) and *in vitro* (Mizuno and Oomura, 1984; Himmi et al., 1996; Dallaporta et al., 1999). In addition to containing these glucoresponsive cells, NST also receives input regarding peripheral glucose levels by way of glucosensors located in hepatic portal/mesenteric vein, which innervate NST via the hepatic branch of the vagus nerve (Niijima, 1984; Adachi et al., 1995; Grabauskas et al., 2010). Furthermore, NST also receives descending inputs from hypothalamic nuclei such as the lateral and paraventricular nuclei that are associated with glucose homeostasis (Marty et al., 2007; Geerling et al., 2010; Biag et al., 2012). Due to this confluence of signals regarding glucose availability as well as its inputs to pre-autonomic nuclei, the NST plays a predominate role in central control of glucose homeostasis (Adachi et al., 1995; Grill and Hayes, 2012).

The NST is also commonly associated with the physiological response to hypoglycemia. These autonomic counter-measures, often referred to collectively as the counter-regulatory response (CRR) include increases in serum levels of glucagon and stress hormones, increased food intake, and an overall increase in sympathetic tone. Systemic administration of the glucoprivic compound 2-deoxy-glucose (2-DG; a glucose analog commonly used to invoke the CRR) results in increased c-Fos expression in the NST (Ritter et al., 1998; Briski and Marshall, 2000; Sanders and Ritter, 2000; Dodd et al., 2010). Focal administration of a similar glucoprivic agent, 5-thio-D-glucose, directly into the NST of rats also drives CRRs such as increased food intake (Ritter et al., 2000) and increases in serum glucagon and stress hormone levels (Andrew et al., 2007).

In addition to its role in glucose homeostasis and hypoglycemic counter-regulation, the NST plays a critical role in a number of other autonomic functions such as regulation of cardiovascular, respiratory, and gastrointestinal reflexes (Blessing, 1997). There is increasing evidence that chemodetection by brainstem astrocytes can drive changes in these autonomic reflexes (Hermann and Rogers, 2009; Hermann et al., 2009; Gourine et al., 2010; Kasymov et al., 2013). Interestingly there is also strong evidence for astrocytic involvement in the autonomic response to hypoglycemia. Systemic administration of the selective glial toxin, methionine sulfoximine, blocks 2-DG induced c-Fos expression in NST (Young et al., 2000). In addition, transgenic mice which only express the type II glucose transporter (GLUT2) in pancreatic beta cells (i.e., expression of GLUT2 was knocked out centrally), demonstrate defects in the CRR (Burcelin and Thorens, 2001). However, these defects are rescued by the selective CNS re-expression of GLUT2 in astrocytes, but not neurons (Marty et al., 2005). Therefore, the aim of the present study was to investigate whether NST-astrocytes respond to physiologically relevant decreases in glucose availability, *in vitro*, as well as to the presence of the glucoprivic compound 2-DG.

## Methods

### Animals

All experimental procedures were conducted under the approval of the Pennington Biomedical Research Center's Institutional Animal Care and Use Committee. Long Evans rats of either sex (body weight between 150 and 250 g) were used in these studies. All animals were housed in a temperature controlled room under 12 h light/dark cycle and provided water and food *ad libitum*. All experimental protocols were performed according to the guidelines set forth by the National Institutes of Health.

### *In vivo* pre-labeling of NST and harvest of hindbrain slices

**Figure 1:**
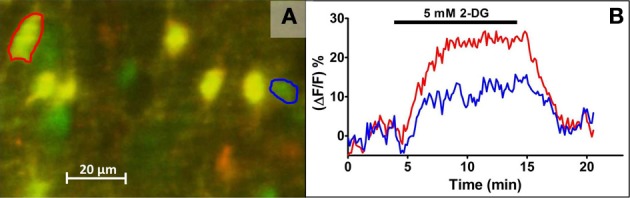
**Live cell calcium imaging of NST astrocytes in response to glucoprivic challenge.** *In vivo* co-injection of the calcium indicator dye, Calcium Green 1-AM (CAG; green) and the glial vital stain sulforhodamine 101 (SR101; red) allows for simple discrimination of astrocytes and neurons in the *in situ* brain stem slice preparation. Following a dual exposure utilizing the 488 nm and 561 nm laser lines, a composite image is obtained in which astrocytes are clearly labeled yellow, i.e., positive for both CAG and SR101, while neurons remain green **(A)**. Time-lapse imaging with only the 488 nm laser line can then be used to follow changes in intracellular calcium concentrations, indicated by a proportional change in the intensity of the CAG signal, before, during, and after the application of glucoprivic or low glucose challenges. Regions of interest (ROI) were drawn around an individual neuron (blue circle) and astrocyte (red circle) in **(A)** for analysis of their time-lapse responses to 2-DG challenge **(B)**. Exposure time to 2-DG is represented by bar over graph.

Animals (*N* = 22) were deeply anesthetized with urethane (1.5 g/kg, ip; ethyl carbamate, Sigma) and placed in a stereotaxic frame. Using aseptic technique, the occipital plate of the skull was removed to expose the medullary brainstem. A micropipette 30 micron tip diameter; filled with 0.2% Calcium Green 1 AM (CAG; Life Technologies), 0.3% sulforhodamine 101 (SR101; Sigma Chemical) and 20% pluronic-DMSO (F-127, in pH 7.2 tris-PBS buffer) was directed toward the medial solitary nucleus using a stereotaxic carrier. Four injections (40 nL each) of the CAG-SR101 solution were made unilaterally into the NST at the level of calamus and 0.2, 0.4, and 0.6mm anterior to calamus; all at a depth of 300 microns below the surface. This injection pattern labeled the entire ipsilateral medial NST (Hermann et al., 2009). CAG, a calcium reporter dye, is taken up by both neurons and glia while SR101 labels only astrocytes (Nimmerjahn et al., 2004; McDougal et al., 2011). SR101 does not interfere with CAG fluorescence (Hermann and Rogers, 2009). Thus, despite the similarity in size of NST neuronal and astrocytic cell bodies, this method made it possible to easily discriminate between these two cell types in the slice preparation (Figure 1). After a 30 min interval to allow for dye uptake, the anesthetized rat was decapitated and the brainstem was quickly harvested. The caudal brainstem was glued to an aluminum block and placed in cold (~4°C) carboxygenated (95% O_2_; 5% CO_2_) cutting solution (recipe below). Coronal sections (300 micron thick) were cut through the medulla using a Leica VT1200 tissue slicer equipped with a sapphire knife (Delaware Diamond Knives). The NST is easily identified in these slices due to its lack of myelin; 4–5 slices containing NST were collected from each animal and placed in normal Krebs' solution (recipe below) which was bubbled with 95% O_2_/5% CO_2_ and maintained at a constant temperature of 29°C. Slices were allowed to equilibrate to these conditions for an hour prior to imaging.

### *In vitro* drugs and solutions

All solutions were freshly prepared on the day of the experiment. The cutting solution contained 110mM choline chloride, 25mM NaHCO_3_, 2.5mM KCl, 7mM MgSO_4_–7H2O, 1.25mM NaH_2_PO_4_, 10mM glucose, and 0.5mM CaCl_2_–2H_2_O. The normal Krebs' solution contained 124mM NaCl, 25mM NaHCO_3_, 3.0mM KCl, 1mM MgSO_4_–7H_2_O, 1.5mM NaH_2_PO_4_, 5mM glucose, and 1.5mM CaCl_2_–2H_2_O. Composition of the “low glucose challenge” solutions was identical to the normal Krebs' solution except that the concentration of glucose was decreased from 5 to 2.5mM, 1mM, or 0.5mM. Given that extracellular brain glucose levels are ~30% that of plasma glucose (Silver and Erecinska, 1994), the range of normal extracellular brain glucose levels runs between 0.5 and 2.5mM (Song et al., 2001). However, brain areas such as the arcuate nucleus of the hypothalamus or the NST, which border circumventricular organs, are predicted to be exposed to glucose levels normally seen in the CSF (3.4mM) or serum (5.5mM) (Levin et al., 2004). Therefore, we include 5mM glucose as our “normal” glucose concentration for comparisons.

The “glucoprivic challenge” solution consisted of 5mM 2-deoxy-D-glucose (2-DG; Sigma-Aldrich, St. Louis, MO) dissolved in normal 5mM glucose Krebs' solution. The osmolarity of the normal Krebs', low glucose challenge, and glucoprivic challenge solutions were adjusted to 300 ± 3 mOsm with sucrose. Tetrodotoxin (1 μM TTX; Sigma-Aldrich, St Louis, MO) was included in some low glucose challenge solutions. Bath application of TTX has been shown to block approximately 95% of the direct neuronal activation of NST astrocytes (McDougal et al., 2011).

### Live cell calcium imaging

Slices were transferred to a custom imaging chamber (Rogers and Hermann, 2012b); perfused at a rate of 2.5 mL/min with normal Krebs' maintained at a temperature of 32°C. Slices were imaged with a Nikon F1 fixed stage upright microscope equipped with a Nikon Fast Scan laser confocal head and a Luca EMCCD camera (Andor Technology, South Windsor, CT). Co-injection of CAG and SR101 allows for discrimination of astrocytes and neurons at the cellular level by comparing images captured using the 488 and 561 nm laser lines (McDougal et al., 2011). That is, at 488 nm, both astrocytes and neurons pre-labeled with CAG will appear green; at 561 nm, all astrocytes will also appear red. A single, dual exposure image was collected just prior to and following each experimental trial in order to confirm the cell types being recorded (Figure 1).

Changes in intracellular calcium concentrations within CAG pre-labeled NST-astrocytes and NST-neurons in response to different stimuli were recorded using the 488 nm laser line to excite the CAG. Increases in intracellular calcium concentrations are reflected as an increase in fluorescence and is indicative of increased cellular activity. During experimental trials, time-lapsed images of mixed fields of NST-astrocytes and neurons were monitored for their responses to low glucose or glucoprivic conditions.

### Experimental design

Each slice was exposed to a single “experimental trial” which consisted of three perfusion conditions of equal duration (i.e., 10 min for each condition). Slices were perfused with normal Krebs' containing 5mM glucose during the initial and final blocks of time. The intervening block was the experimental condition [e.g., “time control” consisting of continued perfusion with 5mM glucose Krebs', low glucose (i.e., 0.5, 1.0, or 2.5mM) or glucoprivic challenge (i.e., 5mM 2-DG with 5mM glucose Krebs')]. Thus, each cell functioned as its own control and transitional differences in activity due to changes in available glucose (i.e., decreasing or increasing) could also be monitored.

In order to rule out the possibility that neuronal synaptic input to glia was responsible for the astrocytic responses to low-glucose conditions, tetrodotoxin (TTX) was used to block action potential-mediated communication between neurons and astrocytes (McDougal et al., 2011). For this study, we specifically chose the 1mM glucose challenge, to ensure that the neurons would not be compromised by very low glucose. In these experiments, TTX (1 μM) was present during all three perfusion periods [i.e., normal (5mM), low glucose challenge (1mM), and normal (5mM) glucose]. The responses of cells during the low glucose challenge plus TTX were then compared to responses of cells exposed to an identical experimental trial that did not use TTX, i.e., identical blocks of 0.5, 1 and 5mM profusion conditions without TTX.

Time-lapse images were analyzed using Nikon NIS-Elements software. Each cell in a field was delineated as a region of interest (ROI) and the average intensity of all pixels within each ROI was calculated for each time point (Figure 1). These data were exported to Microsoft Excel for analysis offline.

Relative changes in intracellular calcium in response to all stimulation paradigms were quantified as percent changes in fluorescence: (Δ *F*/*F*)%, where *F* is the baseline fluorescence intensity within an area of interest (e.g., the outline of an SR101-labeled astrocyte) before stimulation, and Δ *F* is the change from this value resulting from stimulus-induced cellular activity (Helmchen, 2000). Background fluorescence (i.e., non-responsive areas in same field) was subtracted from both *F* and Δ *F*. An individual cell was considered to have responded to a given stimulus if the stimulus induced a peak change in (Δ *F*/*F*)% > 5% (O'Malley et al., 2006; Rogers et al., 2006a,b; Hermann et al., 2009; McDougal et al., 2011).

Parameters including average baseline fluorescence intensity, peak intensity, as well as the onset of the response to the low glucose or glucoprivic challenge were determined for each ROI. These values were used to calculate: (a) latency to onset of response, (b) peak response magnitude (percent increase in intensity from baseline), (c) response offset (time at which the response magnitude decayed to 1/*e* of the peak value; ~37%), and (d) response magnitude (area under the curve from response onset to response offset) for each ROI. In order to verify the macro-defined values, each was displayed graphically on a line plot of intensity vs. time and visually inspected for accuracy.

The criteria of low-glucose (or 2DG) responder types:

- *Positive*: Peak response > 5% and AUC > 175 (Δ*F*/*F*)% minute and Δ*F*/*F* values returned to 1/*e* of peak (~37%) before the end of the trial.
- *Negative*: Peak response < –5% and AUC< −175 (Δ*F*/*F*)% minute
- *Sustained activation*: Peak response > 5% and Δ*F*/*F* values failed to return to 1/*e* of peak (~37%) before the end of the trial. AUC is undefined in these cells because there is no “stop time” which is defined as the time at which Δ*F*/*F* values returned to 1/*e* of peak (~37%).
- *Non-responders*: Any cell which did not meet the above criteria.

### Data analysis

The cell type (i.e., astrocyte or neuron) represented by each ROI was determined by the presence or absence of SR101 staining. The average values of three separate response parameters (peak, latency, and response magnitude) were determined for each cell type in each experimental condition, and then compared statistically. Astrocytic response parameters across the three concentrations of glucose were compared to neuronal response parameters using two-way analysis of variance (i.e., cell type vs. concentration of glucose). Separate analyses were made of the average response parameters of astrocytes vs. neurons to a glucoprivic condition (i.e., 5mM glucose plus 5mM 2-DG) using unpaired *t*-tests.

Studies using TTX to determine if astrocytic responses were secondary to neuronal activation used unpaired *t*-tests to evaluate cellular response parameters of astrocytes or neurons during the 1mM glucose challenge with vs. without TTX in the perfusion solution. All statistical analyses were performed using GraphPad Prism software (GraphPad Software, La Jolla, CA); *p*-values < 0.05 were considered statistically significant.

## Results

### A subset of NST-astrocytes and neurons respond to low glucose challenge

**Figure 2:**
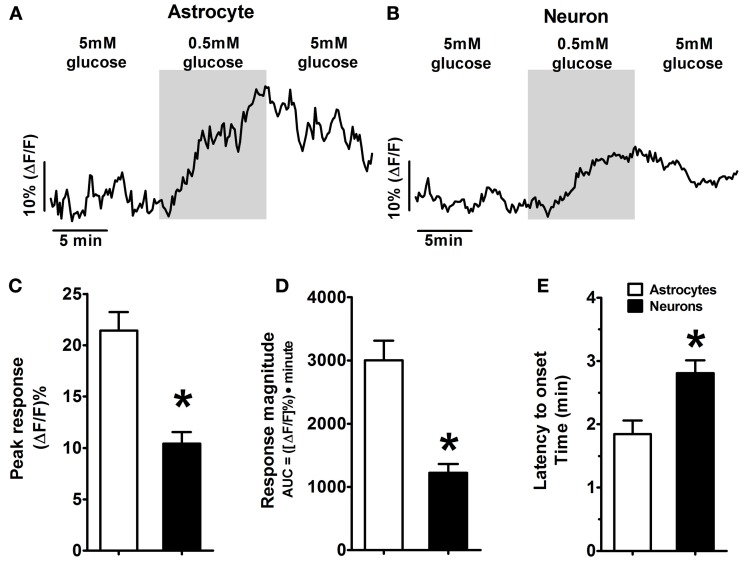
**Example of response patterns of low glucose-positive astrocytes and neurons in the hindbrain slice preparation to low glucose challenge.** As seen in Figure 1, mixed fields of NST-astrocytes and neurons were recorded using confocal live cell calcium imaging. Changes in intracellular calcium levels were reflected as changes in intensity of the fluorescent calcium indicator dye, Calcium Green 1-AM. Both low glucose-positive **(A)** astrocytes and **(B)** neurons were activated in response to a change in the extracellular glucose concentration from 5 to 0.5mM; this activation returned toward baseline levels following return to 5mM glucose. [Traces in **(A,B)** are the average response of five representative low glucose positive astrocytes and neurons, respectively]. Two-Way ANOVA: **(C)** Peak response and **(D)** response magnitude demonstrated significant main effects attributed to the cell type (*p* < 0.05) as well as significant main effects attributed to the concentration of glucose (*p* < 0.05) (see also Figure 3). These main effects are qualified due to the significant interactions between cell types and glucose concentrations. Thus, NST-astrocytes were more responsive than NST-neurons to low glucose conditions particularly at the 0.5mM concentration. **(E)** Latency to onset of response to low glucose also demonstrated a significant main effect attributed to cell type (*p* < 0.05); astrocytes responded at 1.7 ± 0.2 min (mean ± s.e.m.) compared to neurons at 2.6 ± 0.2 min. ^*^P < 0.05.

Brainstem slices challenged by conditions of low glucose availability (i.e., bath application of Krebs' solution containing 0.5, 1.0, or 2.5mM glucose) showed fluxes in intracellular calcium levels in both NST-astrocytes and neurons. The cellular responses were measured by changes in the fluorescent intensity of CAG relative to individual baseline levels. The relative changes in cytoplasmic calcium were expressed as percent changes in fluorescence [(Δ*F/F*)%] of the CAG reporter dye, where *F* is the intensity of the baseline fluorescence signal before stimulation, and Δ*F* is the difference between the peak fluorescence intensity and the baseline signal (Figures 2, 4, 5).

Both NST-astrocytes and neurons displayed one of four distinct response patterns as a result of exposure to each low glucose challenge (Table 1). The most prevalent response pattern was the “non-responder” which showed no response to conditions of low glucose availability (47% of astrocytes; 51% of neurons). The second largest category encountered was a “low glucose positive” response pattern where cells showed an increase in intracellular calcium concentrations when exposed to low glucose and baseline levels of intracellular calcium concentrations returned following the return to 5mM glucose Krebs' solution (39% of astrocytes; 37% of neurons) (Figures 2A,B; Table 1). The remaining cells responded either with a decrease in intracellular calcium levels and were characterized as “low glucose negative” (13% of astrocytes; 11% of neurons) or displayed an increase in intracellular calcium signaling which failed to return to baseline levels following the return to 5mM glucose levels. This last group is referred to as “sustained activation” (1% of astrocytes; 1% of neurons; Table 1).

**Table 1:** **Classification of cellular response patterns to low glucose (top) or glucoprivic (bottom) challenges**.

	**Low glucose positive (%)**	**Low glucose negative (%)**	**Low glucose sustained (%)**	**Low glucose NR (%)**
Astrocytes (263 total)	39	13	1	47
Neurons (160 total)	37	11	1	51
	**2DG positive (%)**	**2DG negative (%)**	**2DG sustained (%)**	**2DG NR (%)**
Astrocytes (118 total)	42	8	3	47
Neurons (81 total)	26	16	6	52

### Response to low glucose challenge differed based on cell type and glucose concentration

**Figure 3:**
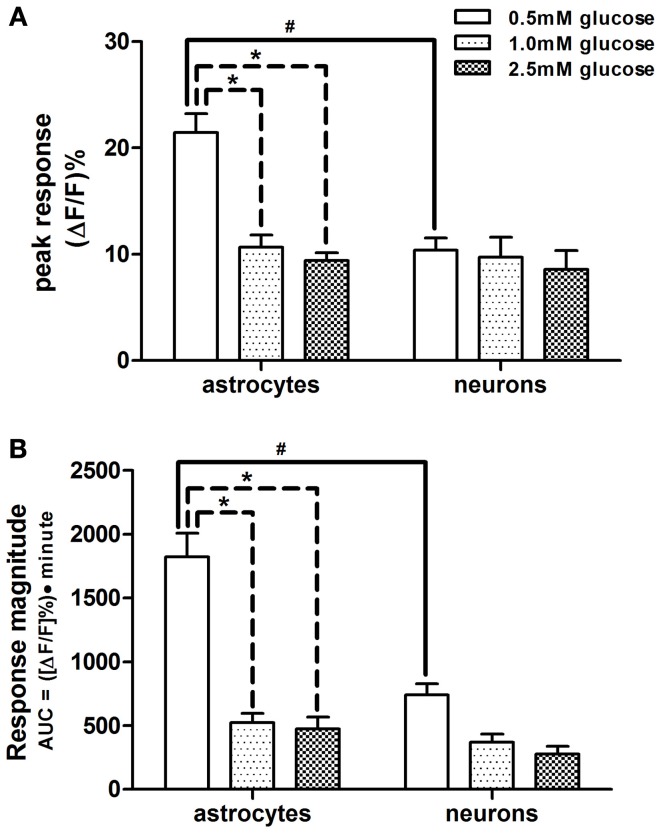
**Comparison of response parameters of NST-astrocytes and -neurons to low glucose conditions.** Subsets of both astrocytes and neurons responded with increased activation, as seen by their change in fluorescence, when exposed to lower glucose concentrations in the perfusion solution. Two-Way ANOVA of the peak and magnitude of astrocytic and neuronal responses to low glucose conditions revealed that astrocytes were more responsive than neurons in **(A)** peak response and **(B)** response magnitude, i.e., area under the curve [significant main effect of cell type; *F*_(1, 159)_ = 8.36 and 12.57, respectively; *p* < 0.05]. There were significant main effects attributed to the concentration of glucose [*F*_(2, 159)_ = 8.40 and 18.57; respectively; *p* < 0.05]. These main effects are qualified due to the significant interactions between cell types and glucose concentrations [*F*_(2, 159)_ = 5.24 and 4.99, respectively; *p* < 0.05]. Bonferroni *post-hoc t*-tests demonstrated that astrocytes and neurons differ significantly in these response parameters when 0.5mM glucose was in the perfusion bath; #*p* < 0.05. In astrocytes, these response parameters at the 0.5mM glucose level where significantly different from those at the 1.0 and 2.5mM glucose levels; ^*^p < 0.05.

Three response parameters were measured in “low glucose positive” astrocytes and neurons following exposure to each low glucose challenge condition (0.5, 1.0, or 2.5mM); see Figure 3 and Table 2. These parameters included: (1) response latency (time between onset of challenge and response onset), (2) response magnitude (area under the curve from response onset to response offset), and (3) peak response (percent increase in CAG intensity from baseline). Two-Way ANOVA revealed that latency to onset of response demonstrated a significant main effect attributed to cell type [*F*_(1, 111)_ = 6.38, *p* < 0.05]. Latency to onset of response to low glucose for astrocytes was 1.7 ± 0.2 min (mean ± SEM) compared to neurons at 2.6 ± 0.2 min. Peak response and response magnitude also demonstrated significant main effects attributed to the cell type [*F*_(1, 159)_ = 8.36 and 12.57, respectively; *p* < 0.05] as well as significant main effects attributed to the concentration of glucose [*F*_(2, 159)_ = 8.40 and 18.57; respectively; *p* < 0.05]. These main effects are qualified due to the significant interactions between cell types and glucose concentrations [*F*_(2, 159)_ = 5.24 and 4.99, respectively; *p* < 0.05]. Thus, astrocytes were more responsive than neurons to low glucose conditions particularly at the 0.5mM concentration (Figure 3).

**Table 2:** **Response parameters of NST astrocytes and neurons responsive to low-glucose or 2DG conditions**.

**Condition**	**Peak response (DF/F)%**		**Magnitude of response (Area under the curve = change over time)**		***N***	
	**Astrocytes**	**Neurons**	**Astrocytes**	**Neurons**	**Astrocytes**	**Neurons**
5mM 2DG	15.5 ± 1.1	14.0 ± 1.0	2329 ± 228	2093 ± 229	56	21
0.5mM glucose	21.4 ± 1.8	10.4 ± 1.1	3005 ± 308	1224 ± 140	46	33
1.0mM glucose	10.1 ± 1.1	9.3 ± 1.8	678 ± 124	391 ± 60	35	17
2.5mM glucose	8.4 ± 0.7	7.8 ± 1.5	704 ± 139	391 ± 94	29	12

Bonferroni *post-hoc t*-tests showed that astrocytes and neurons differ in these response parameters to 0.5mM glucose in the perfusion bath (Figure 2). Additionally, astrocytes demonstrated significantly larger peak responses and response magnitudes to 0.5mM glucose compared to either 1.0 or 2.5mM glucose challenges (Figure 3).

### A subset of NST-astrocytes and neurons respond to glucoprivic challenge

**Figure 4:**
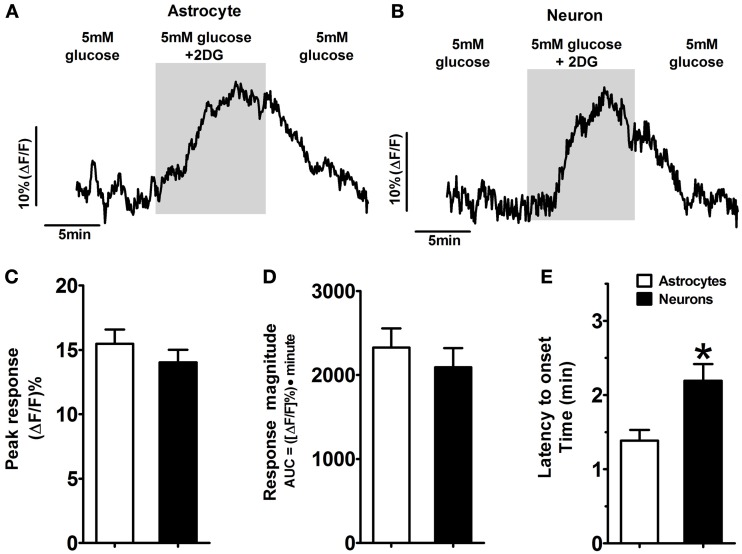
**Activated response patterns of astrocytes and neurons in the hindbrain slice preparation to glucoprivic challenge (i.e., 2-DG-positive).** Approximately 40% of NST-astrocytes and 25% of NST-neurons responded to a glucoprivic solution consisting of 5mM 2-Deoxy-D-glucose (2-DG) dissolved in media containing normal (5mM) glucose concentrations with an increase in activity (Tables 1, 2). Examples of 2-DG-positive astrocytes and neurons activated in response to the glucoprivic challenge are shown [Traces in **(A,B)** are the average response of five representative 2-DG-positive astrocytes and neurons, respectively]. The peak responses **(C)** and response magnitudes **(D)** were similar in both astrocytes and neurons. However, these two cell types differed in time to onset of response **(E)**. NST-astrocytes averaged 1.4 ± 0.1 min compared to the onset of NST-neuronal response at 2.2 ± 0.2 min (^*^p < 0.05).

NST-astrocytes and neurons showed similar response patterns during glucoprivic challenge where 5mM 2-DG was included in the 5mM glucose-Krebs' solution (Figure 4). Most astrocytes (47%) and neurons (52%) were “non-responders” (Table 1). The next largest class was “2-DG positive” which displayed an increase in intracellular calcium signaling during exposure to this glucoprivic condition that returned to baseline following washout (42% of astrocytes; 26% of neurons). The remainder of cells could be characterized as either “2-DG negative” (8% of astrocytes; 16% of neurons) or “sustained activation” (3% of astrocytes; 6% of neurons). Unpaired *t*-test comparisons revealed that astrocytes had significantly shorter latencies to response onset compared to neurons [1.4 ± 0.1 min vs. 2.2 ± 0.2 min; *t*_(73)_ = 3.00; *p* = 0.0037]. There were no differences in peak response (15.5 ± 1.1% vs. 14.0 ± 1.0%; astrocytes vs. neurons) or response magnitude (2329 ± 228 vs. 2093 ± 229) between astrocytes and neurons during glucoprivic challenge (Table 2).

### Low glucose signaling in NST-astrocytes is independent of neuronal signaling

**Figure 5:**
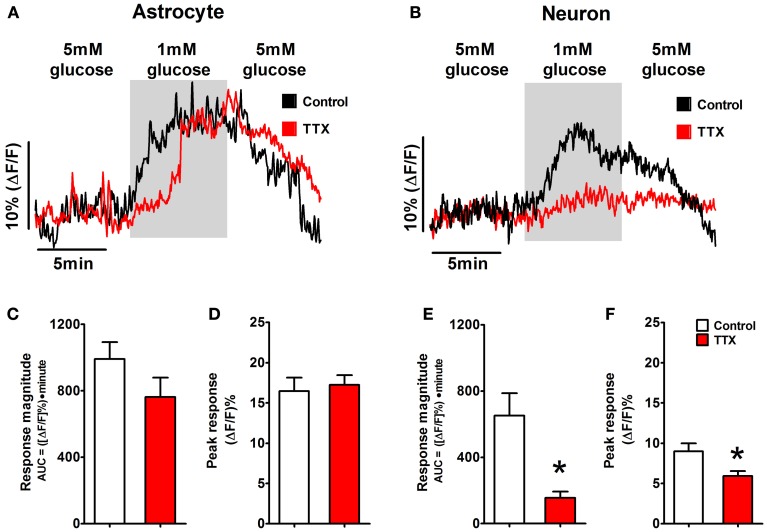
**Astrocytic glucodetection is independent of neuronal glucodetection.** Mixed fields of NST-astrocytes and neurons were exposed to 1mM glucose concentrations with and without the presence of TTX in the perfusion bath. **(A)** The response of NST-astrocytes to a low glucose challenge is shown by black trace. The presence of 1 μM TTX did not effectively alter the astrocytic response (red trace). In contrast, **(B)** the response of NST-neurons to a 1mM low glucose challenge was nearly abolished in the presence of bath TTX (black trace = control condition; red trace = TTX condition). Specifically, TTX did not produce any statistically significant changes in astrocytic **(C)** response magnitude or **(D)** peak response to low glucose conditions. In contrast, TTX caused a statistically significant reduction in both neuronal response magnitude **(E)** and peak responsiveness **(F)** when compared to control neuronal responses to low glucose. These results suggest that activation of NST-astrocytes by low glucose challenge is independent of neuronal gluco-sensation. NST neuronal calcium signaling in response to low glucose challenge, on the other hand, may be dependent on other neuronal inputs or on modulation of the voltage-dependent sodium conductance. [^*^p < 0.05; unpaired *t*-test comparing response parameters in control vs. TTX condition. Traces in **(A,B)** are the average response of five representative low glucose positive astrocytes and neurons, respectively.]

In order to rule out the possibility that neuronal synaptic input to glia was responsible for the astrocytic responses to low-glucose conditions, TTX was used to block action potential-mediated communication between neurons and astrocytes (Figure 5). Astrocytic peak responsiveness to a 1mM low glucose challenge was unaffected by TTX (15.3 ± 0.9% vs. 17.5 ± 1.7%), although response magnitude was somewhat lower than control levels (991 ± 102 vs. 763 ± 116; Figures 5A,C,**D**). The slight reduction in the size of the astrocytic responses in the presence of TTX likely represents a loss of tonic neuronal activation of NST-astrocytes independent of glucose availability, possibly mediated by glutamate release from vagal afferents (McDougal et al., 2011).

In contrast, neuronal peak response and response magnitude during the same low glucose challenge were significantly reduced by TTX [peak response: 9.0 + 1.0% vs. 6.2 + 0.6%; *t*_(17)_ = 2.422, *p* = 0.013; magnitude: 652 ± 135 vs. 156 ± 37; *t*_(17)_ = 3.043, *p* = 0.004 (Figures 5B,E,F)]. NST neuronal calcium signaling in response to low glucose challenge may be dependent on other neuronal inputs or on modulation of the voltage-dependent sodium conductance.

## Discussion

The dorsal medulla and NST have long been associated with the central detection of the glucose availability and the control of the glycemic state (Bernard, 1855; Marty et al., 2007; Grill and Hayes, 2012). Recent evidence is emerging in support of astrocytic involvement in glucose homeostasis (Young et al., 2000; Guillod-Maximin et al., 2004; Marty et al., 2005). Our present report provides evidence that astrocytes in the NST respond to changes in glucose availability. Following exposure to conditions of low glucose, a subset of NST-astrocytes and neurons exhibit an increase in intracellular calcium signaling, this is reversed following restoration of normal glucose availability. Both the peak and magnitude of the astrocytic response varied across different levels of low glucose availability. The size of the neuronal response across the different glucose concentrations did not vary significantly. In comparison to adjacent neurons, astrocytes displayed larger peak responses and response magnitudes, with a reduced latency to respond, following exposure to 0.5mM glucose concentrations. Note that the activation of astrocytes by reduced glucose was not a smooth linear function; increased activation occurred abruptly at 0.5mM glucose. This behavior may be a reflection of the operation of an emergency low threshold detection mechanism of the sort driving counter-regulation in response to hypoglycemia (Song et al., 2001; Marty et al., 2007). Furthermore, this astrocytic response to low glucose challenge was independent of neuronal activation, as it was preserved during bath application of TTX. Note that bath application of TTX nearly eliminated the neuronal response to the same low glucose challenge. This suggests that low glucose-mediated increases in neuronal cytoplasmic calcium are produced as a consequence of action potential discharge (Gobel and Helmchen, 2007). In addition, a subset of NST-astrocytes and neurons exhibited an increase in intracellular calcium signaling in response to bath application of the glucoprivic compound 2-DG (5mM). The onset of this response to glucoprivation was more rapid in astrocytes as compared to adjacent neurons. However, the latencies we observed for astrocytes and neurons are similar to those reported previously for glucose sensitive cells in the hypothalamus or hindbrain (Song et al., 2001; Kang et al., 2004; Balfour et al., 2006). These observations suggest that NST-astrocytes are important sensors of low glucose levels, and that the chemodetection of glucose availability by NST-astrocytes may play an important role in the autonomic regulation of glucose homeostasis.

Astrocyte chemodetection is emerging as an important means by which the brain mediates autonomic homeostasis. Astrocytes in the ventral medulla increase cytoplasmic calcium in response to elevated pCO_2_. This increase in glial calcium triggers a gliotransmission mechanism that, in this instance, causes the release of ATP onto adjacent respiratory rhythmogenic neurons (Gourine et al., 2010; Marina et al., 2013). Our own work has shown that astrocytes in the NST are activated by proteinase activated receptor (PAR) agonists and, in turn, release glutamate onto adjacent NST neurons (Hermann et al., 2009). In addition to glucoregulatory functions, the NST also suppresses gastric motility through vago-vagal reflex connections with parasympathetic motor neurons (Rogers and Hermann, 2012a). This mechanism may explain in part why bleeding trauma and burn injuries (both conditions generate large amounts of PAR-activating thrombin) are associated with profound gastrointestinal stasis (Hermann et al., 2009). It remains to be determined whether similar glial-neural interactions are present between glucosensitive astrocytes and NST neurons.

In addition, the mechanism by which a *reduction* in glucose availability signals an *increase* in cytoplasmic calcium in the NST-astrocyte also remains indeterminate. While astrocytic expression of GLUT2 appears necessary for proper glucose counter-regulation (Marty et al., 2005), the transduction mechanisms often coupled to GLUT2 produce changes in calcium that run in the *opposite direction* to that observed for astrocytes. For example, pancreatic beta cells respond with an increase in intracellular calcium levels in response to *increasing* extracellular glucose. Here, glucose is transported through the GLUT2 transporter and metabolized via glycolysis and the tricarboxcylic acid cycle to CO_2_, producing ATP. Increased cellular ATP energy charge acts to phosphorylate and inactivate K_*ATP*_ channels, leading to depolarization, activation of voltage gated calcium channels and ending in excitation—secretion coupling (Schuit et al., 2001; Holsbeeks et al., 2004; Levin et al., 2004).

However, others have also reported that glucose restriction in cultured astrocytes causes an *increase* in astrocytic cytoplasmic calcium signals (Arnold, 2005). Similar to our present observations, this increase in cytoplasmic calcium is reversed with the restoration of normal levels of glucose in the perfusion bath. There is evidence suggesting that astrocytes are highly dependent on glycolysis for ATP production; removal of glucose or blocking glucose utilization may rapidly starve astrocytes of glucose for ATP production (Kahlert and Reiser, 2000). With impaired glycolysis and subsequent low ATP following low extracellular glucose availability, the calcium-ATPase pump in the endoplasmic reticulum (ER) of astrocytes fails and ER calcium is released to the cytoplasm (Kahlert and Reiser, 2000; Arnold, 2005). While these results are consistent with our observations in the slice preparation, it is not clear if this mechanism is actually responsible for the cytoplasmic calcium signal seen by our laboratory in *in situ* NST-astrocytes. For example, the response time for astrocytes in our NST slice preparation is about five-fold faster than that reported for cultured hippocampal astrocytes.

One possibility for the acceleration of this mechanism relative to cultured astrocytes would be the expression of GLUT2 and glucokinase (GK) by glucosensitive NST-astrocytes. Glial expression of both GLUT2 and GK has been reported previously (Garcia et al., 2003; Arluison et al., 2004; Young and McKenzie, 2004; Millan et al., 2010), as well as the specific expression of GLUT2 by a subset of NST-astrocytes (Leloup et al., 1994). Co-expression of these proteins would permit cytoplasmic glucose to flow backward through the GLUT2 transporter following a reduction in extracellular glucose below intracellular glucose concentrations, thus speeding the elimination of fuel for glycolysis, and therefore accelerating ER calcium release. Further studies utilizing single cell qPCR could be employed to directly investigate whether GLUT2 or GK is preferentially expressed by glucosensitive NST-astrocytes as compared to non-glucoresponsive NST astrocytes.

Another possibility which could account for the more rapid astrocytic cytoplasmic calcium signals in our preparation is that NST-astrocytes may contain a much more active low-glucose detection mechanism via direct signaling of low glucose availability by GLUT2. GLUT2 is the only mammalian glucose transporter known to act as a “transceptor,” i.e., a protein which functions as both a transporter and receptor. The receptor function of mammalian GLUT2 is mediated by the large intracellular loop between the 6th and 7th transmembrane domains (Guillemain et al., 2000). Transgenic mice generated to knock down a GLUT2-intracellular loop domain yielded animals that demonstrated an inability to detect glucose but left the GLUT2-dependent glucose transport unaffected (Stolarczyk et al., 2007, 2010). Depression of glucose detection in these animals resulted in increased daily food intake due to increased meal sizes.

The transporter function of GLUT2 has also been linked to the activation of hepatic transcription factor, a carbohydrate response-element binding protein (Uyeda and Repa, 2006). However, it seems unlikely that similar GLUT2-mediated transcriptional regulation in NST-astrocytes could bring about the large and rapid changes in intracellular calcium signaling we observed in response to low glucose availability. Instead, it seems more likely that there may be a direct interaction between the intracellular loop domain of GLUT2 and signaling pathways present in NST-astrocytes; although, at present, there is no information available as to how it might function.

### Conflict of interest statement

The authors declare that the research was conducted in the absence of any commercial or financial relationships that could be construed as a potential conflict of interest.

## References

[B1] Adachi A. Kobashi M. Funahashi M. (1995). Glucose-responsive neurons in the brainstem. Obes. Res. 3 Suppl. 5, 735S–740S 10.1002/j.1550-8528.1995.tb00493.x 8653556

[B2] Andrew S. F. Dinh T. T. Ritter S. (2007). Localized glucoprivation of hindbrain sites elicits corticosterone and glucagon secretion. Am. J. Physiol. Regul. Integr. Comp. Physiol. 292, R1792–1798 10.1152/ajpregu.00777.2006 17218439

[B3] Arluison M. Quignon M. Nguyen P. Thorens B. Leloup C. Penicaud L. (2004). Distribution and anatomical localization of the glucose transporter 2 (GLUT2) in the adult rat brain–an immunohistochemical study. J. Chem. Neuroanat. 28, 117–136 10.1016/j.jchemneu.2004.05.009 15482899

[B4] Arnold S. (2005). Estrogen suppresses the impact of glucose deprivation on astrocytic calcium levels and signaling independently of the nuclear estrogen receptor. Neurobiol. Dis. 20, 82–92 10.1016/j.nbd.2005.02.002 16137569

[B5] Balfour R. H. Hansen A. M. Trapp S. (2006). Neuronal responses to transient hypoglycaemia in the dorsal vagal complex of the rat brainstem. J. Physiol. 570, 469–484 10.1113/jphysiol.2005.098822 16284073 PMC1479878

[B6] Bernard C. (1855). Lecons de Physiologie Experimentale Appliques á la Medecine. Paris: Bailliere

[B7] Biag J. Huang Y. Gou L. Hintiryan H. Askarinam A. Hahn J. D. (2012). Cyto- and chemoarchitecture of the hypothalamic paraventricular nucleus in the C57BL/6J male mouse: a study of immunostaining and multiple fluorescent tract tracing. J. Comp. Neurol. 520, 6–33 10.1002/cne.22698 21674499 PMC4104804

[B8] Blessing W. W. (1997). Anatomy of the lower brainstem, in The Lower Brainstem and Bodily Homeostasis, (Oxford: Oxford University Press), 29–100

[B9] Briski K. P. Marshall E. S. (2000). Caudal brainstem Fos expression is restricted to periventricular catecholamine neuron-containing loci following intraventricular administration of 2-deoxy-D-glucose. Exp. Brain Res. 133, 547–551 10.1007/s002210000448 10985689

[B10] Burcelin R. Thorens B. (2001). Evidence that extrapancreatic GLUT2-dependent glucose sensors control glucagon secretion. Diabetes 50, 1282–1289 10.2337/diabetes.50.6.1282 11375328

[B11] Cottrell G. T. Ferguson A. V. (2004). Sensory circumventricular organs: central roles in integrated autonomic regulation. Regul. Pept. 117, 11–23 10.1016/j.regpep.2003.09.004 14687696

[B12] Dallaporta M. Himmi T. Perrin J. Orsini J. C. (1999). Solitary tract nucleus sensitivity to moderate changes in glucose level. Neuroreport 10, 2657–2660 10.1097/00001756-199908200-00040 10574387

[B13] Dodd G. T. Williams S. R. Luckman S. M. (2010). Functional magnetic resonance imaging and c-Fos mapping in rats following a glucoprivic dose of 2-deoxy-D-glucose. J. Neurochem. 113, 1123–1132 10.1111/j.1471-4159.2010.06671.x 20236391

[B14] Garcia M. Millan C. Balmaceda-Aguilera C. Castro T. Pastor P. Montecinos H. (2003). Hypothalamic ependymal-glial cells express the glucose transporter GLUT2, a protein involved in glucose sensing. J. Neurochem. 86, 709–724 10.1046/j.1471-4159.2003.01892.x 12859684

[B15] Geerling J. C. Shin J. W. Chimenti P. C. Loewy A. D. (2010). Paraventricular hypothalamic nucleus: axonal projections to the brainstem. J. Comp. Neurol. 518, 1460–1499 10.1002/cne.22283 20187136 PMC2868510

[B16] Gobel W. Helmchen F. (2007). *In vivo* calcium imaging of neural network function. Physiology (Bethesda). 22, 358–365 10.1152/physiol.00032.2007 18073408

[B17] Gourine A. V. Kasymov V. Marina N. Tang F. Figueiredo M. F. Lane S. (2010). Astrocytes control breathing through pH-dependent release of ATP. Science 329, 571–575 10.1126/science.1190721 20647426 PMC3160742

[B18] Grabauskas G. Song I. Zhou S. Owyang C. (2010). Electrophysiological identification of glucose-sensing neurons in rat nodose ganglia. J. Physiol. 588, 617–632 10.1113/jphysiol.2009.182147 20008464 PMC2828136

[B19] Grill H. J. Hayes M. R. (2012). Hindbrain neurons as an essential hub in the neuroanatomically distributed control of energy balance. Cell Metab. 16, 296–309 10.1016/j.cmet.2012.06.015 22902836 PMC4862653

[B20] Gross P. M. Wall K. M. Pang J. J. Shaver S. W. Wainman D. S. (1990). Microvascular specializations promoting rapid interstitial solute dispersion in nucleus tractus solitarius. Am. J. Physiol. 259, R1131–R1138 2260724 10.1152/ajpregu.1990.259.6.R1131

[B21] Guillemain G. Loizeau M. Pincon-Raymond M. Girard J. Leturque A. (2000). The large intracytoplasmic loop of the glucose transporter GLUT2 is involved in glucose signaling in hepatic cells. J Cell Sci 113(Pt 5), 841–847 10671373 10.1242/jcs.113.5.841

[B22] Guillod-Maximin E. Lorsignol A. Alquier T. Penicaud L. (2004). Acute intracarotid glucose injection towards the brain induces specific c-fos activation in hypothalamic nuclei: involvement of astrocytes in cerebral glucose-sensing in rats. J. Neuroendocrinol. 16, 464–471 10.1111/j.1365-2826.2004.01185.x 15117340

[B23] Helmchen F. (2000). Calibration of fluorescent calcium indicators, in Imaging Neurons: A Laboratory Manual, eds Yuste R. Lanni F. Konnerth A. (Cold Spring Harbor, NY: Cold Spring Harbor Laboratory Press), 32.31–32.39

[B24] Hermann G. E. Rogers R. C. (2009). TNF activates astrocytes and catecholaminergic neurons in the solitary nucleus: implications for autonomic control. Brain Res. 1273, 72–82 10.1016/j.brainres.2009.03.059 19348788 PMC2693276

[B25] Hermann G. E. Van Meter M. J. Rood J. C. Rogers R. C. (2009). Proteinase-activated receptors in the nucleus of the solitary tract: evidence for glial-neural interactions in autonomic control of the stomach. J. Neurosci. 29, 9292–9300 10.1523/JNEUROSCI.6063-08.2009 19625519 PMC2773000

[B26] Himmi T. Dallaporta M. Perrin J. Orsini J. C. (1996). Neuronal responses to delta 9-tetrahydrocannabinol in the solitary tract nucleus. Eur. J. Pharmacol. 312, 273–279 10.1016/0014-2999(96)00490-6 8894609

[B27] Holsbeeks I. Lagatie O. Van Nuland A. Van De Velde S. Thevelein J. M. (2004). The eukaryotic plasma membrane as a nutrient-sensing device. Trends Biochem. Sci. 29, 556–564 10.1016/j.tibs.2004.08.010 15450611

[B28] Kahlert S. Reiser G. (2000). Requirement of glycolytic and mitochondrial energy supply for loading of Ca(2+) stores and InsP(3)-mediated Ca(2+) signaling in rat hippocampus astrocytes. J. Neurosci. Res. 61, 409–420 10.1002/1097-4547(20000815)61:4<409::AID-JNR7>3.0.CO;2-M 10931527

[B29] Kang L. Routh V. H. Kuzhikandathil E. V. Gaspers L. D. Levin B. E. (2004). Physiological and molecular characteristics of rat hypothalamic ventromedial nucleus glucosensing neurons. Diabetes 53, 549–559 10.2337/diabetes.53.3.549 14988237

[B30] Kasymov V. Larina O. Castaldo C. Marina N. Patrushev M. Kasparov S. (2013). Differential sensitivity of brainstem versus cortical astrocytes to changes in pH reveals functional regional specialization of astroglia. J. Neurosci. 33, 435–441 10.1523/JNEUROSCI.2813-12.2013 23303924 PMC3690976

[B31] Leloup C. Arluison M. Lepetit N. Cartier N. Marfaing-Jallat P. Ferre P. (1994). Glucose transporter 2 (GLUT 2): expression in specific brain nuclei. Brain Res. 638, 221–226 10.1016/0006-8993(94)90653-X 8199863

[B32] Levin B. E. Routh V. H. Kang L. Sanders N. M. Dunn-Meynell A. A. (2004). Neuronal glucosensing: what do we know after 50 years? Diabetes 53, 2521–2528 10.2337/diabetes.53.10.2521 15448079

[B33] Marina N. Tang F. Figueiredo M. Mastitskaya S. Kasimov V. Mohamed-Ali V. (2013). Purinergic signalling in the rostral ventro-lateral medulla controls sympathetic drive and contributes to the progression of heart failure following myocardial infarction in rats. Basic Res. Cardiol. 108, 317 10.1007/s00395-012-0317-x 23187902 PMC3540348

[B34] Marty N. Dallaporta M. Foretz M. Emery M. Tarussio D. Bady I. (2005). Regulation of glucagon secretion by glucose transporter type 2 (glut2) and astrocyte-dependent glucose sensors. J. Clin. Invest. 115, 3545–3553 10.1172/JCI26309 16322792 PMC1297256

[B35] Marty N. Dallaporta M. Thorens B. (2007). Brain glucose sensing, counterregulation, and energy homeostasis. Physiology (Bethesda). 22, 241–251 10.1152/physiol.00010.2007 17699877

[B36] McDougal D. H. Hermann G. E. Rogers R. C. (2011). Vagal afferent stimulation activates astrocytes in the nucleus of the solitary tract via AMPA receptors: evidence of an atypical neural-glial interaction in the brainstem. J. Neurosci. 31, 14037–14045 10.1523/JNEUROSCI.2855-11.2011 21957265 PMC3445261

[B37] Millan C. Martinez F. Cortes-Campos C. Lizama I. Yanez M. J. Llanos P. (2010). Glial glucokinase expression in adult and post-natal development of the hypothalamic region. ASN Neuro 2, e00035 10.1042/AN20090059 20531973 PMC2881537

[B38] Mizuno Y. Oomura Y. (1984). Glucose responding neurons in the nucleus tractus solitarius of the rat: *in vitro* study. Brain Res. 307, 109–116 10.1016/0006-8993(84)90466-9 6147174

[B39] Niijima A. (1984). The effect of D-glucose on the firing rate of glucose-sensitive vagal afferents in the liver in comparison with the effect of 2-deoxy-D-glucose. J. Auton. Nerv. Syst. 10, 255–260 10.1016/0165-1838(84)90021-3 6090521

[B40] Nimmerjahn A. Kirchhoff F. Kerr J. N. Helmchen F. (2004). Sulforhodamine 101 as a specific marker of astroglia in the neocortex *in vivo*. Nat. Methods 1, 31–37 10.1038/nmeth706 15782150

[B41] O'Malley D. Reimann F. Simpson A. K. Gribble F. M. (2006). Sodium-coupled glucose cotransporters contribute to hypothalamic glucose sensing. Diabetes 55, 3381–3386 10.2337/db06-0531 17130483 PMC1948974

[B42] Ritter S. Dinh T. T. Zhang Y. (2000). Localization of hindbrain glucoreceptive sites controlling food intake and blood glucose. Brain Res. 856, 37–47 10.1016/S0006-8993(99)02327-6 10677609

[B43] Ritter S. Llewellyn-Smith I. Dinh T. T. (1998). Subgroups of hindbrain catecholamine neurons are selectively activated by 2-deoxy-D-glucose induced metabolic challenge. Brain Res. 805, 41–54 10.1016/S0006-8993(98)00655-6 9733914

[B44] Rogers R. C. Hermann G. E. (2012a). Brainstem control of gastric function, in Physiology of the Gastrointestinal Tract, 4th Edn., ed Johnson L. R. (San Diego, CA: Elsevier Academic Press), 861–892 10.1016/B978-0-12-382026-6.00031-2

[B45] Rogers R. C. Hermann G. E. (2012b). Tumor necrosis factor activation of vagal afferent terminal calcium is blocked by cannabinoids. J. Neurosci. 32, 5237–5241 10.1523/JNEUROSCI.6220-11.2012 22496569 PMC3342927

[B46] Rogers R. C. Nasse J. S. Hermann G. E. (2006a). Live-cell imaging methods for the study of vagal afferents within the nucleus of the solitary tract. J. Neurosci. Methods 150, 47–58 10.1016/j.jneumeth.2005.05.020 16099514

[B47] Rogers R. C. Van Meter M. J. Hermann G. E. (2006b). Tumor necrosis factor potentiates central vagal afferent signaling by modulating ryanodine channels. J. Neurosci. 26, 12642–12646 10.1523/JNEUROSCI.3530-06.2006 17151266 PMC6674848

[B48] Sanders N. M. Ritter S. (2000). Repeated 2-deoxy-D-glucose-induced glucoprivation attenuates Fos expression and glucoregulatory responses during subsequent glucoprivation. Diabetes 49, 1865–1874 10.2337/diabetes.49.11.1865 11078453

[B49] Schuit F. C. Huypens P. Heimberg H. Pipeleers D. G. (2001). Glucose sensing in pancreatic beta-cells: a model for the study of other glucose-regulated cells in gut, pancreas, and hypothalamus. Diabetes 50, 1–11 10.2337/diabetes.50.1.1 11147773

[B50] Silver I. A. Erecinska M. (1994). Extracellular glucose concentration in mammalian brain: continuous monitoring of changes during increased neuronal activity and upon limitation in oxygen supply in normo-, hypo-, and hyperglycemic animals. J. Neurosci. 14, 5068–5076 8046468 10.1523/JNEUROSCI.14-08-05068.1994 PMC6577171

[B51] Song Z. Levin B. E. McArdle J. J. Bakhos N. Routh V. H. (2001). Convergence of pre- and postsynaptic influences on glucosensing neurons in the ventromedial hypothalamic nucleus. Diabetes 50, 2673–2681 10.2337/diabetes.50.12.2673 11723049

[B52] Stolarczyk E. Guissard C. Michau A. Even P. C. Grosfeld A. Serradas P. (2010). Detection of extracellular glucose by GLUT2 contributes to hypothalamic control of food intake. Am. J. Physiol. Endocrinol. Metab. 298, E1078–E1087 10.1152/ajpendo.00737.2009 20179244

[B53] Stolarczyk E. Le Gall M. Even P. Houllier A. Serradas P. Brot-Laroche E. (2007). Loss of sugar detection by GLUT2 affects glucose homeostasis in mice. PLoS ONE 2:e1288 10.1371/journal.pone.0001288 18074013 PMC2100167

[B54] Uyeda K. Repa J. J. (2006). Carbohydrate response element binding protein, ChREBP, a transcription factor coupling hepatic glucose utilization and lipid synthesis. Cell Metab. 4, 107–110 10.1016/j.cmet.2006.06.008 16890538

[B55] Watts A. G. Donovan C. M. (2010). Sweet talk in the brain: glucosensing, neural networks, and hypoglycemic counterregulation. Front. Neuroendocrinol. 31:32–43 10.1016/j.yfrne.2009.10.006 19836412 PMC2813965

[B56] Yettefti K. Orsini J. C. El Ouazzani T. Himmi T. Boyer A. Perrin J. (1995). Sensitivity of nucleus tractus solitarius neurons to induced moderate hyperglycemia, with special reference to catecholaminergic regions. J. Auton. Nerv. Syst. 51, 191–197 10.1016/0165-1838(94)00130-C 7769152

[B57] Yettefti K. Orsini J. C. Perrin J. (1997). Characteristics of glycemia-sensitive neurons in the nucleus tractus solitarii: possible involvement in nutritional regulation. Physiol. Behav. 61, 93–100 10.1016/S0031-9384(96)00358-7 8976538

[B58] Young J. K. Baker J. H. Montes M. I. (2000). The brain response to 2-deoxy glucose is blocked by a glial drug. Pharmacol. Biochem. Behav. 67, 233–239 10.1016/S0091-3057(00)00315-4 11124386

[B59] Young J. K. McKenzie J. C. (2004). GLUT2 immunoreactivity in gomori-positive astrocytes of the hypothalamus. J. Histochem. Cytochem. 52, 1519–1524 10.1369/jhc.4A6375.2004 15505347 PMC3957823

